# Influenza A (H1H1) in the post-pandemic period: readjusting the focus based on e-health

**DOI:** 10.1186/1753-6561-5-S6-P89

**Published:** 2011-06-29

**Authors:** J Mapril, C Palos, A Pedroso, M Ressureição, C Alves

**Affiliations:** 1 Hospital da Luz, Lisbon, Portugal

## Introduction / objectives

Influenza A (H1N1) pandemics was the 1^st^ in the last 4 decades and consumed important resources. In the post-pandemic period, healthcare facilities had to readjust strategies to optimize resources while ensuring adequate clinical approach of suspect cases and epidemiologic surveillance.

## Methods

Hospital da Luz, a paper-free hospital, developed a plan for initial approach and follow-up of suspect cases, based on the e-health concept, coordinated by the Infection Control Committee (ICC) and in collaboration with the Internal Medicine Department. Approach included risk assessment using a electronic medical registry: “Flu tool” – a questionnaire generating automatic prescriptions of virologic tests for at risk patients, stated indications for admission, stratified patients for severity and generated a notification to the ICC. According to this evaluation, respiratory swabs were obtained for PCR (H1N1) Influenza A test for at risk patients, and antiviral therapy was prescribed while waiting results. The laboratory notified ICC of results of the H1N1 tests and ICC provided patients with test results (by phone call), either suggesting discontinuation of oseltamivir in case of a negative test or clinical reevaluation in all patients with a positive test and risk factors for a complicated ilness. ICC scheduled Internal Medicine outpatient visit within 48h of test result for at risk patients. Inhospital cases were notified by e-mail to the Ministry of Health.

## Results

Every patient was adequately followed.

## Conclusion

Using the e-health concept, Hospital da Luz developed a new strategy for the approach and follow-up of suspect cases with universal assessment and orientation, improving quality of care.

## Disclosure of interest

None declared.

